# Supplementary material for: Physiological and Molecular Responses of Woody Plants Exposed to Future Atmospheric CO2 Levels under Abiotic Stresses
Source: Plants (Basel). 2022 Jul 20;11(14):1880. doi: 10.3390/plants11141880 (PMC9322912; doi:10.3390/plants11141880)
Supplement: Supplementary file 1 [file plants-11-01880-s001.zip › plants-1781594-supplementary.pdf]

Supplementary material

Physiological and molecular responses of woody plants exposed to future atmospheric CO_2 levels under abiotic stresses

Number of Tables: 1

Table S1. Overview of studies with woody species exposed to elevated [CO_2] alone or combined with abiotic stresses. *Studies involving molecular approaches. Description of the abbreviations present in the table: **WUE**: Water use efficiency; **gs**: stomatal conductance; **E**: transpiration rate; **Chl:** Chlorophyll; **ABA**: Abscisic acid; **iWUE:** Intrinsic water use efficiency; **PS:** Photosystem; **GLO**: Glycolate oxidase; **PPP**: Pentose phosphate pathway

**Species**	**e[CO_2]**	**Interaction with other abiotic stress**	**Main effect of e[CO_2] sole or combined with abiotic stress**	**Reference**
Acacia karroo and *Acacia nilotica*	180 - 1000 (1 year, OTC)	No	↑ Photosynthesis, WUE, and overall biomass<br>↓ gs<br>Light photosynthesis acclimation in the second season	[22]
Alnus hirsuta	360 x 720 (>1 year, GC)	Phosphorus and drought	↑ Nodule biomass and N_2 fixation under high P availability	[137]
Anacardium occidentale	380 x 760 (15 days, GC)	Salt	↓ Photosynthesis without affecting g_s and E<br>↓ Photosynthesis, g_s, E, GLO activity and the contents of H_2O_2, NH_4^+, and glyoxylate under salinity<br>↑ NPQ, K^+/Na^+ ratio, and the content of anthocyanin, sucrose, amino acid and total soluble proteins under salinity	[126]
Angiosperm and gymnosperm tree species (57)	340-980	No	↓ g_s according to the evolutionary scale (evergreen gymnosperms < deciduous angiosperms < evergreen angiosperms)	[20]
Aulonemia aristulata	380 x 760 (3 weeks, OTC)	No	↑ Photosynthesis, WUE, and overall biomass<br>↓ gs	[58]
Betula papyrifera	380 x 550 (Long-term, FACE)	O_3	↑ Photosynthesis, specialized compounds, expression of glycolysis-related genes and farnesyl-PP and phytyl-PP<br>↓ Expression of pre-squalene and squalene synthesis genes, and leaf senescence	[135]
Betula papyrifera and *Populus tremuloides*	380 x 560 (Long-term, FACE)	O_3	↑ Photosynthesis, monosaccharides<br>↓ gs, leaf senescence	[136]
Betula pendula	380 x 760 (~ 1 year, OTC)	O_3	↓ Oxidative stress and senescence-related gene expression and proteins.	[135]*
Betula pendula	360 x 720 (3 years, OTC)	O_3	↑ Photosynthesis, leaf dry weight, leaf area<br>↓ Photosynthetic protein content, Chl and PS density	[59]
Betula pendula	360 x 720 (3 years, OTC)	O_3	↑ Photosynthesis, starch, leaf area, stem diameter, biomass, and C/N ratio<br>↓ Total leaf N, Rubisco amount and activity, and leaf senescence	[60,134]
Betulaceae species	370 x 500 (2 years, FACE)	Soil nutrition	↓ Photosynthesis by lower C/N ratio	[56]
Coffea arabica	400 x 700 (7 months, OTC)	Drought	↑ Photosynthesis, WUE, respiration, overall biomass, and root length<br>↓ Photorespiration rates and oxidative pressure	[49]
Coffea arabica	400 x 700 (5 months, OTC)	Drought	↑ Faster stomatal closure, water potential, plant hydraulic conductance, aquaporin gene expression	[112]

			↓ Whole plant transpiration<br>No association with g_s or leaf ABA levels	
Coffea arabica	390 x 600 (4 years, FACE)	No	↑ Photosynthesis, g_s, WUE, growth, and soil carbon concentration<br>↓ Leaf area, diameter of the second-order axes	[25]
Coffea arabica	400 x 550 (4 years, FACE)	Seasonal drought	↑ Photosynthesis in summer and winter, and the concentration of soluble carbohydrates, organic acids, and amino acids in leaves	[68]
Coffea arabica	400 x 760 (44 days, OTC)	Drought	↑ Photosynthesis, WUE, leaf water potential and carbohydrate concentration	[111]
Coffea arabica	400 x 800 (40 days, OTC)	Drought	↑ Photosynthesis, iWUE, leaf concentration of 5-O-caffeoylquinic acid and caffeine	[113]
Coffea arabica	390 x 550 (~2 years, FACE)	No	↑ Photosynthesis<br>↓ Photorespiration<br>No changes in stomatal and mesophyll conductance, leaf nitrogen and phosphorus concentrations	[24]
Coffea arabica	380 x 700 (4.4 years, GC)	Heat	↓ Impact on physical and chemical traits of coffee beans<br>Preserved bean quality	[100]
Coffea arabica and *Coffea canephora*	380 x 700 (1 year; GC)	No	↑ Photosynthesis, WUE, stomatal size, photosynthetic and respiratory proteins<br>↓ Stomatal density<br>No changes in PSII efficiency, non-structural carbohydrates, photosynthetic pigment and membrane permeability	[40]
Coffea arabica and *Coffea canephora*	380 x 700 (7 years, GC)	Drought	↑ Photosynthesis, photosynthetic proteins<br>↓ Energy dissipation and PSII impact<br>No correlation with stomatal closure and leaf ABA level	[114]
Coffea arabica and *Coffea canephora*	380 x 700 (7 years, GC)	Drought	↑ Leaf amino acid concentration in *C. arabica*<br>Little effect in leaf metabolite levels in *C. canephora*	[115]*
Coffea arabica and *Coffea canephora*	380 x 700 (10 months, GC)	Heat	No clear pattern between transcriptomic data and the mitigating role of e[CO_2] suggesting post-transcriptional modifications	[99]*
Coffea arabica and *Coffea canephora*	380 x 700 (10 months, GC)	Heat	↑ Gene expression and activity of glutathione reductase, catalase, Cu- and Zn-superoxide dismutase, ascorbate peroxidase and gene expression of HSP70, ELIPs, and chaperonins<br>↓ Reactive oxygen species and PSII photoinhibition	[98]
Coffea arabica and *Coffea canephora*	380 x 700 (10 months, GC)	Heat	↑ Photosynthesis (photochemical efficiency, energy use and biochemical functioning) and WUE.	[96]
Deciduous trees	FACE	No	↑ Leaf area index, WUE<br>↓ Photosynthesis (except in alder), light compensation point, g_s of most species	[41]
Ecosystem-scale relationship	FACE	No	↑ Plant productivity and N acquisition positive correlation<br>↓ Plant N concentration regardless of productivity	[53]
Eucalyptus forest	400 x 550 (5 years, FACE)	No	↑ N/P ratio in sapwood<br>↓ N concentration and N/P ratio in young leaf<br>No changes in P concentraation	[51]
Eucalyptus saligna	380 x 680 (5 months, GC)	Seasonal drought	↑ Leaf day respiration<br>No changes in seasonal acclimation	[65]
Eucalyptus saligna and *Eucalyptus sideroxylon*	290 x 400 x 650 (~4 months, GC)	Drought and temperature	Contrasting photosynthetic response between genotypes under drought<br>No beneficial effect under elevated temperature and drought	[19]
Eucalyptus species	290 x 400 x 650 (~4 months, GC)	Heat	↑ Photosynthesis, maximal electron transport rate and carboxylation rate, and growth	[93,97]
Eucalyptus tereticornis	400 x 640 (4 months, GC)	Heat x Phosphorus	↑ Total dry biomass, leaf number, leaf area under high P supply	[138]
Forest species	long-term field-based studies	Review	↓ g_s mostly in young, deciduous and water-stressed trees<br>No acclimation of g_s	[35]
Liquidambar styraciflua	Long-term FACE	Seasonal drought	↓ Sap flow, g_s, leaf senescence	[108]
Liquidambar styraciflua	370 x 570 (2 years; FACE)	No	↑ Number of mitochondria, mass per area, and starch in leaves<br>No changes in leaf respiration	[66]
Mature forest	ambient x +150 ppm (FACE)	No	↑ C uptake through gross primary production and soil respiration	[70]

Morus multicaulis Perr.	410/460 x 710/760 ppm (4 months, GC)	No	↑ Plant height, stem diameter, number of leaves, biomass production, and P and K uptake<br>↓ Photosynthesis, g_s, transpiration and concentration of chlorophyll, and N and P in leaves and roots	[52]
Olea europaea	360 x 700 (3 months, GC)	Salt	↑ Photosynthesis, and WUE<br>↓ NaCl content in plant tissue, PSII efficiency and chlorophyll	[125]
(*Populus tomentosa* x *P. bolleana*) x *P. tomentosa*	385x550x720 (3 months; GC)	No	↑ Stem diameter, gibberellic acid, cytokinin zeatin riboside<br>↓ Photosynthesis, abscisic acid<br>DEGs in leaves were involved in metabolic processes (77.55%) and response to stimulus of external environment (14.29%). Aldehyde dehydrogenase and pyruvate kinase were the core interconnected with other genes.	[76]*
Pinus densiflora	350 x 700 (73 days; GC)	Phosphorus	↑ P requirement, and shoot growth<br>No changes in photosynthesis and stomatal conductance	[37]
Pinus halepensis	421 x 867 (18 months, GC)	Heat and drought	↑ Photosynthesis, WUE, root protein stability<br>↓ Respiration<br>Little effect in combined stress	[110]*
Pinus massoniana	400 x 800 (0 to 24 h, GC)	No	↑ Expression of glycolysis, carboxylic acid cycle, PPP pathway, sucrose synthesis, gibberellin, salicylic acid, brassinolide and methyl jasmonate pathways related genes<br>↓ Expression of photosynthetic, and abscisic acid-related genes	[67]*
Pinus radiata	370 x 650 (3 years, OTC)	Nitrogen	↑ Stem basal area in high-N supply, and sapwood density<br>No changes in N status and lignification	[77]
Pinus sylvestris	380 x 580 (5 months, OTC)	Heat and nitrogen	↑ Radial growth, latewood density and maximum intra-ring density, thicker (alone) or thinner (+heat) tracheid walls<br>↓ Cellulose concentration, number of tracheids	[78]
Pinus sylvestris and *Pinus nigra*	390 x 570 (4 months, FACE)	Drought	No changes in starch concentrations, biomass production and mortality	[109]
Pinus taeda	380 x 600 (4 months; FACE)	Seasonal drought	↑ Photosynthesis under eCO_2, except at peak drought<br>No changes in water-saving and g_s	[36]
Populus	380 x 550 (2 years, FACE)	No	↑ Photosynthesis<br>↓ Leaf senescence, leaf area index, and chlorophyll content	[73]
Populus × euramericana	380 x 550 (6 years, FACE)	No	↑ DEGs in young leaves (developmental response related – xyloglucan endotransglycosylase and calcium-signalling)<br>↓ DEGs in semimature leaves (Photosynthesis, chloroplast biogenesis- and function-related)	[61]*
Populus deltoides	400 x 800 x 1200 (3 years; FACE)	No	↑ Leaf area, stem biomass, expression of genes involved with storage proteins, cell wall expansion in leaves and lignin formation and polymerisation or ethylene response factors in stems.	[74]*
Populus deltoides	400 x 800 (3 years, GC)	Drought and VPD	↑ Leaf area, leaf loss under drought, stomatal density<br>↓ Woody density	[107]
Populus tremuloides	372 x 560 (~3 months, FACE)	No	↑ Photosynthesis and stem volume, expression of genes related to active defense/response to stress, carbohydrate/starch biosynthesis and growth in the CO_2-responsive clone, and passive defense (e.g. lignin, phenylpropanoid) and cell wall thickening in the CO_2-unresponsive clone	[62]*
Populus tremuloides	360 x 560 (5 years; FACE)	O_3	↑ Expression chloroplast 30S ribosomal, PSII, and auxin-binding related genes<br>↓ Expression of Rubisco activase genes	[72]*
Populus tremuloides	380 x 560 (~4 months, FACE)	O_3	↓ Chlorophyll and carotenoid concentration, activity of antioxidant enzymes and PAL and expression of 1-aminocyclopropane-1-carboxylic acid (ACC)-oxidase transcript	[21]
Populus x *euramericana*	380 x 550 (2 years, FACE)	No	↑ Pathways for secondary metabolism (anthocyanin biosynthesis) and glycolysis during senescence, LDOX (leucoanthocyanidin dioxygenase) and DFR (dihydroflavonol reductase) related transcripts	[75]*
Populus x *euramericana*	370 x 550 (Long-term, FACE)	No	↑ Cell expansion and production, basipetal gradient of leaf development	[81]
(*Quercus mongolica* and *Kalopanax septemlobus*) (*Betula maximowicziana* and *Acer mono*)	370 x 500 (4 years, FACE)	No	↑ Plant height, photosynthesis and vessel area in *K. septemlobus* and vessels in all species, except *B. maximowicziana* and number of cambial cells in two ring-porous species	[57]

Quercus rubra	380 x 700 (4 months, GC)	Heat and drought	↑ Photosynthesis and biomass in heatwave	[139]
